# Analysis of RNA-Seq datasets reveals enrichment of tissue-specific splice variants for nuclear envelope proteins

**DOI:** 10.1080/19491034.2018.1469351

**Published:** 2018-10-06

**Authors:** Charlotte Capitanchik, Charles R. Dixon, Selene K. Swanson, Laurence Florens, Alastair R. W. Kerr, Eric C. Schirmer

**Affiliations:** a The Wellcome Centre for Cell Biology and Institute of Cell Biology, University of Edinburgh, Edinburgh, UK; b Stowers Institute for Medical Research, Kansas City, MO, USA

**Keywords:** Tissue-specific, nuclear membrane, proteomics, muscular dystrophy, nuclear envelopathies, splice variant

## Abstract

Laminopathies yield tissue-specific pathologies, yet arise from mutation of ubiquitously-expressed genes. A little investigated hypothesis to explain this is that the mutated proteins or their partners have tissue-specific splice variants. To test this, we analyzed RNA-Seq datasets, finding novel isoforms or isoform tissue-specificity for: Lap2, linked to cardiomyopathy; Nesprin 2, linked to Emery-Dreifuss muscular dystrophy and Lmo7, that regulates the Emery-Dreifuss muscular dystrophy linked emerin gene. Interestingly, the muscle-specific Lmo7 exon is rich in serine phosphorylation motifs, suggesting regulatory function. Muscle-specific splice variants in non-nuclear envelope proteins linked to other muscular dystrophies were also found. Nucleoporins tissue-specific variants were found for Nup54, Nup133, Nup153 and Nup358/RanBP2. RT-PCR confirmed novel Lmo7 and RanBP2 variants and specific knockdown of the Lmo7 variantreduced myogenic index. Nuclear envelope proteins were enriched for tissue-specific splice variants compared to the rest of the genome, suggesting that splice variants contribute to its tissue-specific functions.

## Introduction

Nuclear envelope (NE) links to inherited disease yielded the conundrum of how mutations in widely expressed proteins can yield many distinct pathologies, each focused in different tissues [1]. The NE is a double membrane system comprised of inner (INM) and outer (ONM) nuclear membranes and associated proteins that must integrate all communication between the nucleus and the rest of the cell and tissue. Most trafficking of proteins, nucleotides, and small molecules through the NE is directed by the nuclear pore complexes (NPCs), built from >30 nucleoporin (nup) proteins together with a host of transport factors [2]. Less used mechanisms also exist for membrane fusion [3,4] and autophagy [5,6]: factors required for both mechanisms were identified in NE proteomics studies [7–10]. Signals can also be passed through mechanotransduction [11–13], which relies on connections between cytoplasmic filament systems and the intermediate filament lamin nucleoskeleton underlying the inner surface of the INM. The primary proteins making the connections are SUN and nesprin proteins, respectively INM and ONM NE transmembrane proteins (NETs) that form the linker of nucleoskeleton and cytoskeleton (LINC) complex [14,15]. Other INM NETs directly connect to chromatin and directly participate in genome regulation [16,17].

Mutations leading to human disease have been found in all these NE components. Mutations in many different nucleoporins lead to particular cancers each affecting a particular tissue [18]. Some nucleoporin mutations also cause tissue-specific inherited diseases such as Triple A syndrome from Aladin and infantile bilateral striatal necrosis from Nup62 [19,20]. Over 400 different mutations in the *LMNA* gene, encoding nucleoskeletal component lamin A, cause a wide spectrum of debilitating tissue-specific monogenic disorders despite that *LMNA* is near-ubiquitously expressed. These range from several muscular dystrophies and cardiomyopathy to dermopathy, neuropathies, and several lipodystrophies [21,22]. Similarly, despite their ubiquitous expression, both the SUN and nesprin LINC complex components are mutated in Emery-Dreifuss muscular dystrophy [23,24] and nesprin mutations also cause autosomal recessive cerebellar ataxia, a brain-specific neuropathy [25]. The INM NETs emerin and LAP1 are linked to muscular dystrophies while LBR and MAN1 are linked to bone disorders [26–29]. At the same time, LBR also is linked to the blood disorder Pelger-Huet anomaly [30]. One hypothesis for how tissue-specificity in pathology is achieved with the aforementioned mutations in widely expressed proteins is that the mutations disrupt binding to tissue-specific interaction partners. Previously identification of tissue-specific NETs focused on the level of expression, with hundreds of NETs restricted in expression to specific tissues being discovered through proteomics of NEs extracted from muscle, liver, and blood [7–10]. Additionally, some NPC components have differential expression such as Nup358 in skeletal myogenesis [31], Nup133 in mouse embryonic development [32], and Nup210 in muscle and neuronal differentiation [33,34].

Tissue-specific alternative pre-mRNA splicing presents another mechanism to generate tissue-specific NE proteins. *LMNA* is spliced to encode principally lamin A and lamin C, the relative levels of which vary between cell types though both are near ubiquitously expressed [35]. There are also two rare but widely expressed splice variants: lamin A∆10 and progerin [36]. Progerin is principally expressed in patients with Hutchison-Gilford Progeria Syndrome and is reported to be expressed in normal aging [37]. Though some NETs have many known splice variants such as Lap2 with at least six (α, β, Υ, δ, ϵ, ζ) [38–40], these have not been investigated in detail for their tissue-specificity of expression. There are two exceptions to this. First, many tissue-specific NE protein splice variants have been reported for testis including lamins B3 (spliced from the *LMNB1* gene) and C2 (spliced from the *LMNA* gene), and SUN variants [41–44]. The other exception is the nesprin family: several tissue-specific splice variants have been reported for both the larger nesprins 1 and 2. Known isoforms of nesprin-1 range in size from 53 kDa to >1,000 kDa, with the shortest isoforms being expressed exclusively in human cardiac and skeletal muscle tissues [45,46]. More recently, siRNA depletion of distinct SUN1 splice forms has been shown to either increase or decrease breast cancer cell migration depending on the isoform depleted [47]. Furthermore, mutations linked to muscular dystrophies are located near to a variable region of the protein that is partially excluded in the SUN1_888 isoform [47]. Critically, defects in the splicing of NE genes can cause disease [48–50].

Identifying tissue-specific splice variants of these proteins is important because different variants can have distinct partners and cellular localizations. Functional studies of NE proteins typically rely on exogenous expression of the more widely expressed isoforms, so that important functional differences in tissue-specific isoforms would be missed. Though several large easily searchable databases exist for tissue-specific gene and protein expression [51–54], the best resource for predicted splice isoforms is the Vertebrate Alternative Splicing and Transcription Database (VastDB). VastDB contains information about splicing events collected from 1,478 RNA-Seq datasets covering human, mouse, and chicken across different tissues, cell lines, and developmental contexts [51]. However, identification of alternately spliced transcripts by RNA-Seq does not indicate whether they are actually translated. Efforts were previously engaged to find support for novel splice variants identified by RNA-Seq in mass spectrometry data, but have been met with limited success. We postulated that our tissue-specific datasets of the NE organelle would have sufficient depth to increase the likelihood of identifying peptides covering splice junctions and set out to test this. Though mostly finding first protein evidence for previously annotated splice variants, the analysis identified several novel tissue-specific splice variants. Furthermore, testing specific splice variants in mouse C2C12 myogenesis confirmed their presence and their knockdown yielded aberrations in myogenesis.

## Results

### NE genes are enriched in alternative splicing

We previously determined the NE proteome from rat liver and muscle and human blood and found that most NE proteins are tissue-restricted [7–10]. While much of the tissue-specificity observed correlated at the level of expression [8], several proteins were also observed that were expressed widely, but only targeted to the NE in certain tissues [55]. One possible explanation for such proteins is that the tissue with unique NE localization of the protein also expressed a unique tissue-specific variant carrying a NE targeting sequence. A large number of spectra generated in mass spectrometry studies are not matched to peptides. This is generally attributed to non-unique sequences or unknown, or not searched for, post-translational modifications, but it could also be explained by tissue-specific splice variants that are not annotated in the databases so that the sequences are not searched for in the mass spectrometry analysis. The likelihood that there are many as yet not annotated splice isoforms is underscored by the increasing number of annotated transcripts with each major new annotation release (Supplemental Figure S1A). Accordingly, we sought to search for evidence of tissue-specific splicing of NE proteins.

To find such splice variants we engaged a workflow (Supplemental Figure S1B) to first identify all transcripts containing different splice junctions in RNA-Seq data. As most of our mass spectrometry data was in rat, it was important to match this to RNA-Seq data from rat, which was missing from VastDB. Therefore, we analyzed RNA-Seq data from five rat tissues (heart, skeletal muscle, liver, brain, and testes) produced by two separate research groups, hereby referred to as GSE4 and GSE5 [54,56]. GSE4 has higher read coverage over junctions, but only one suitable sample per tissue, while GSE5 has lower coverage but many replicates. Novel junctions identified in both datasets were considered to be high confidence. Differentially spliced transcripts were determined and quantified using Modeling Alternative Junction Inclusion Quantification (MAJIQ) software. Splice junctions rather than whole isoforms were quantified due to the known limitations in reconstructing whole transcripts from short read data [57,58]. The software builds splice graphs from RNA-Seq reads spanning spliced junctions, incorporates un-annotated junctions, and uses information from replicate samples to build a Bayesian posterior distribution, outputting the expected percentage spliced in values (E(PSI)) corresponding to the percentage of transcripts that are expected to contain the given splice junction or in the case of a comparison, change in PSI (E(dPSI)). In accordance with previous literature, we considered an alternative splicing event between tissues when E(dPSI) ≥ 0.2.

Datasets from multiple labs were examined such that the identification of novel isoforms could be as robust as possible. As we were particularly interested in myogenesis splice variants due to the links of the NE to multiple muscular dystrophies, we also analyzed three mouse C2C12 myogenesis RNA-Seq datasets from three different labs with multiple replicates. These were considered similar enough based on clustering in principle component analysis (PCA) that they could be combined and analyzed together using MAJIQ (Supplementary Figure S2).

**Figure 1.**
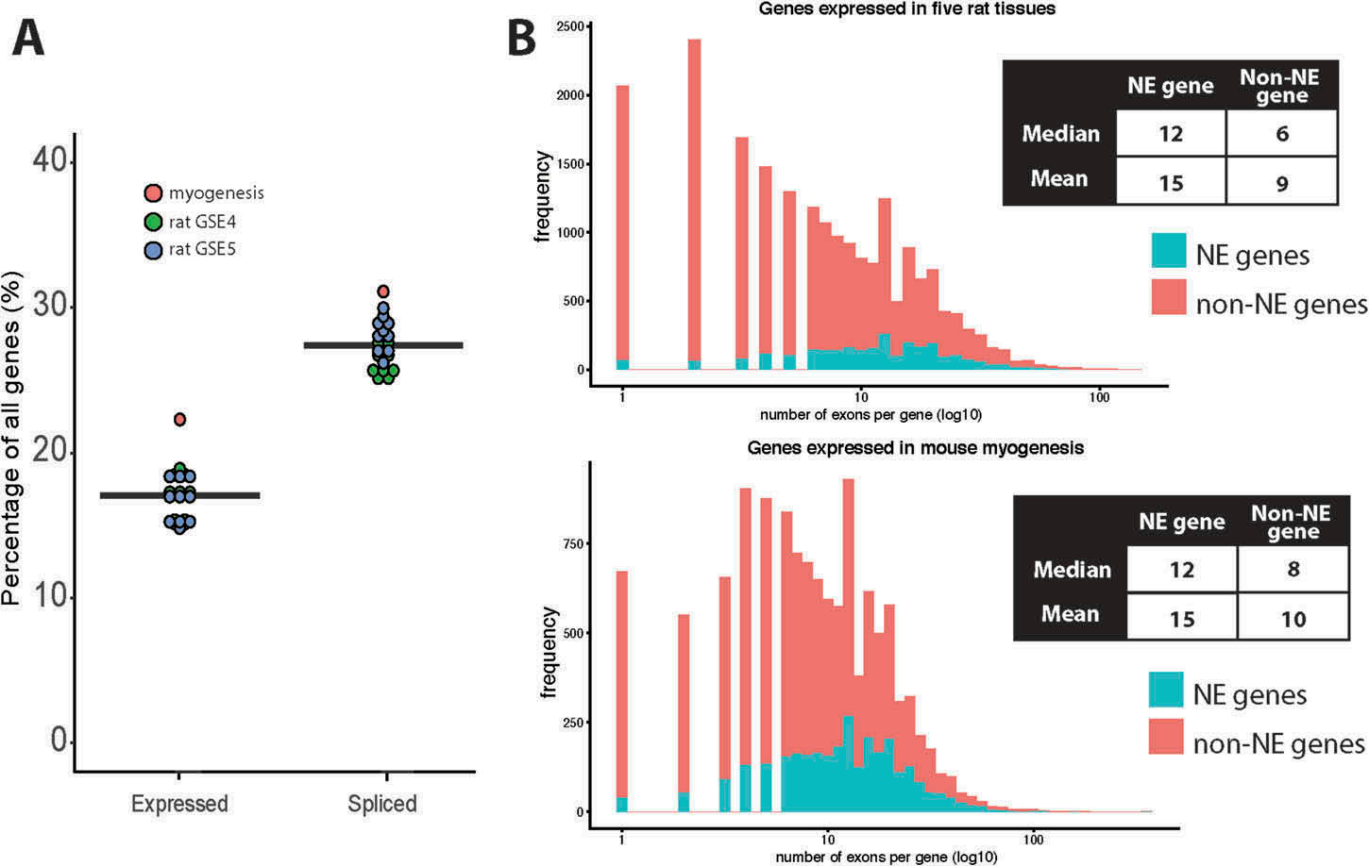
Enrichment of alternatively spliced variants in NE genes. (a) The proportion of expressed genes whose protein products are found at the NE was compared to the proportion of spliced genes encoding a NE protein. Each dot represents a comparison between two tissues or developmental stages, for example muscle vs. liver or myoblast vs. myotube. To determine expressed genes the union of all genes considered to be expressed in both conditions was taken. Whether a gene was expressed was determined in two different ways (described in the methods), but here just the variance stabilizing transformation (VST) method is shown as it gave a more conservative estimate. To determine spliced genes, all genes that contained a differential splicing event with E(dPSI) ≥ 0.2 between the two conditions were taken, as calculated with MAJIQ. (b) The maximum exon number per gene was compared for NE genes vs. non-NE genes and it was found that NE genes have more exons on average than other expressed genes in myogenesis and five rat tissues. Note that only genes expressed in either mouse myogenesis or in the five rat tissues studied were considered, as otherwise the plots were skewed towards genes with very low exon numbers.

Considering alternative splicing to be associated with E(dPSI) ≥ 0.2 and using a subset of NE proteomics datasets that was enriched in NEs over an endoplasmic reticulum (ER)-enriched fraction, 1947/3487 (56%) mouse NE mRNAs were alternatively spliced across the developmental transitions and tissue types studied. 1461/2963 (49%) rat NE mRNAs were alternatively spliced in the union set of the two five-tissue rat sets analyzed, with 327 of these genes being identified in both experiments. To our surprise, genes encoding NE proteins were enriched in alternative splicing between tissues in both rat datasets and in the C2C12 myogenesis model system (Figure 1(a)). NE genes represent 17% of all expressed genes on average, but are over-represented in genes differentially spliced between tissues, comprising 27.5% on average between datasets (Kolmogorov-Smirnov Test, p-value < 0.0001).

Enriched alternative splicing might relate to the number of exons, and therefore available splice sites, in a gene. The number of annotated exons in NE genes versus non-NE genes was examined to see if there was a clear distinction. This analysis was performed for NE genes versus non-NE genes expressed during myogenesis and for the five tissue rat sets. It was important to perform the comparison against expressed genes and not all annotated genes because the NE genes by definition of being identified in proteomics screens are expressed. Information from each gene was downloaded from Ensembl BioMart, and the transcript with the highest number of exons for each expressed gene was considered. On average, NE protein encoding genes had more exons than the average for non-NE protein encoding genes in the genome (Figure 1(b)).

Previous studies demonstrated that alternatively spliced exons are enriched in intrinsically disordered regions of proteins [59,60]. Although prediction algorithms for intrinsic disorder are still in their infancy for high confidence and high-throughput analysis, it has been suggested for yeast NETs that intrinsic disorder is a common property [61]. Searching for intrinsic disorder using IUPred and filtering for ≥ 20 amino acid stretches of disorder against the longest rat sequence available in Ensembl revealed the presence of intrinsically disordered regions in 44% (1283/2913) rat NE proteins compared to 34% for the rest of the proteins encoded by the genome. While this was highly significant by counts (Fisher’s Exact Test, p-value < 0.0001), using the alternative hypothesis that the true odds ratio is not equal to 1 with a 95% confidence interval gives only a slightly higher odds ratio of 1.302.

### Searching NE-enriched proteomics datasets for splice junction peptides

Tissue-specific junction sequence databases were generated for rat muscle and liver tissues. In each case the union of all splice junctions detected by STAR aligner for each replicate from GSE4 and GSE5 was taken. Junctions were filtered to remove junctions supported by less than 6 reads (a threshold adopted from [62,63]) and junctions predicting an intron length of less than 60 nucleotides (a standard cut-off as there are very few smaller annotated junctions) (Supplementary Figure S3). Junction coordinates were extended by 66 nucleotides in both directions, and then translated in three frames according to the directionality of the gene [62,63]. This produced peptide sequences of ~44 amino acids in length. Sequences were removed if the translation produced a stop codon before the junction based on standard practice [62,63]. Novel exons and intron retentions predicted by MAJIQ were similarly translated in three frames and added to the database, but were removed if the translated sequence was less than 7 amino acids long. All novel and annotated junctions were combined with novel exons, intron retentions, and Ensembl rat protein database sequences to produce a final protein sequence database. Finally, coordinates of junction peptides were checked against the original fasta sequence to be sure that the peptide crossed the junction with a start ≤22 amino acids away and an end ≥22 amino acids away.

In total, by re-analyzing our previously described NE-enriched proteomic datasets obtained from liver and muscle, we found 183 unique junction-spanning rat peptides that support 84 un-annotated rat junctions. Of these, 74 junctions occurred in regions of the rat transcriptome that are not annotated to genes; however, the corresponding sequences were annotated in human and mouse transcriptomes and more than half (44 of 74) corresponded to the titin gene. The remaining 8 previously not annotated rat junctions occurred in rat annotated genes for Atl3, Camk2a, Mta1, Rbm7, Ryr1, Safb2, Top2b, and LAP2; however, all of these junctions had been previously annotated in human or mouse.

**Figure 2.**
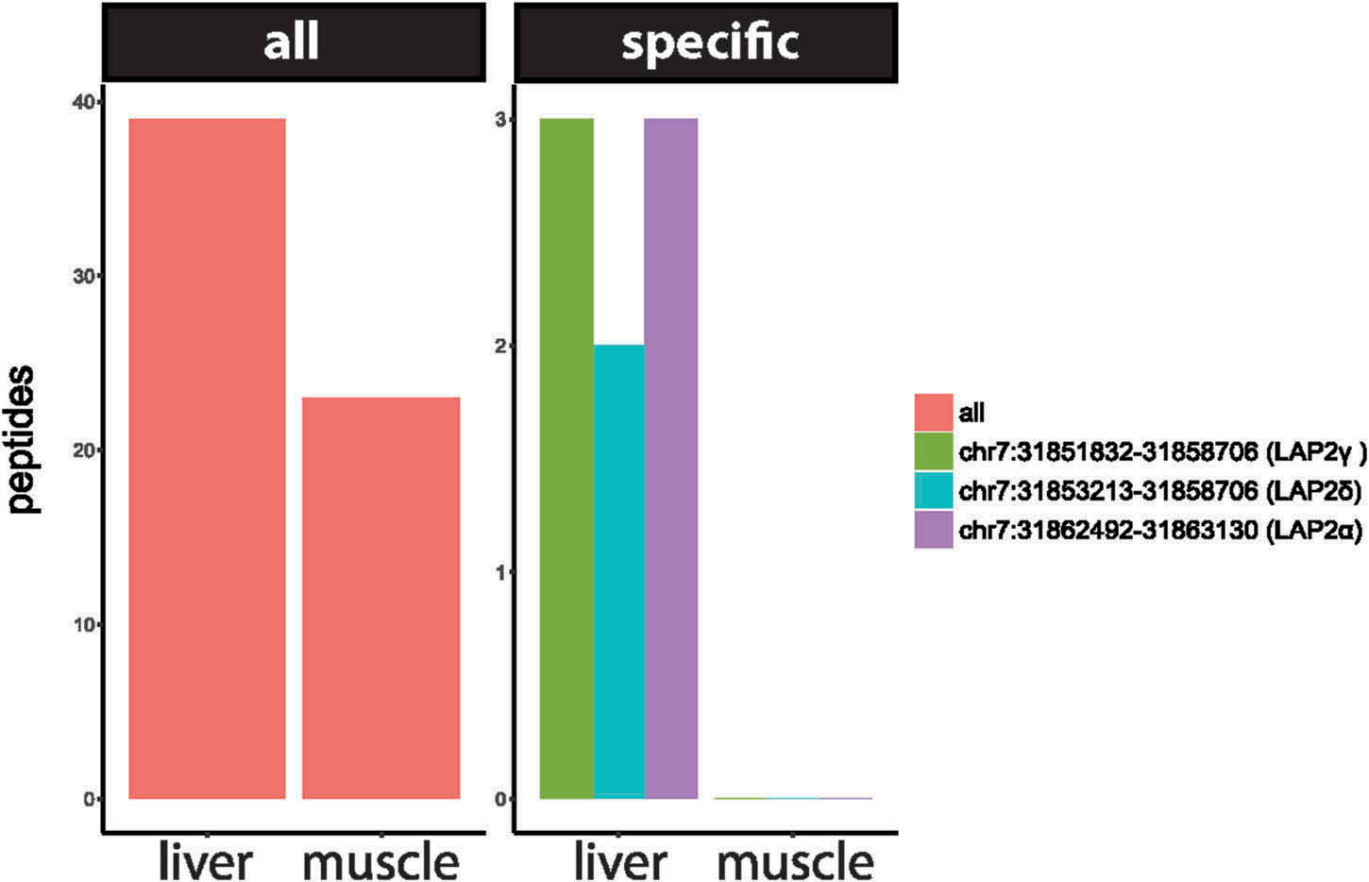
Peptide support for Lap2, showing a similar number of peptides over the whole protein in liver and muscle, but evidence for specific isoforms comes from liver samples only.

While the identification of novel splice variants detected by this method was lower than we had anticipated, looking at the tissue dataset specificity of several splice junction peptides suggested that LAP2α, LAP2δ and LAP2γ are preferentially expressed in rat liver and not muscle (Figure 2). In contrast, for other regions of LAP2 39 peptides were identified in liver and 23 peptides in muscle, suggesting that overall levels and coverage of the total isoform pool was similar. The possible tissue-specific expression of LAP2 isoforms in rat liver could potentially have relevance to human disease because there are several laminopathy links to liver. For example, a laminopathy case caused by a heterozygous R133L Lamin A mutation presented as a generalized lipodystrophy with hepatic steatosis [64] and several other laminopathies, particularly lipodystrophies, present with hepatic distress. Most significantly, LAP2 itself has recently been linked to nonalchoholic fatty liver disease [65].

### Tissue-specific NE gene junctions are discovered in multiple independent RNA-Seq datasets

Although we found evidence for the translation of many splice variants, the peptide coverage for most proteins was much lower than for proteins such as LAP2, thus significantly lowering the probability of recovering the less abundant splice junction peptides. Thus, while the presence of splice junction peptides supports the presence of the translated product, the absence of splice junction peptides is not evidence of the absence of the protein product. Accordingly, we searched for tissue-specificity of possible novel splice variants using just the RNA-Seq data.

**Figure 3.**
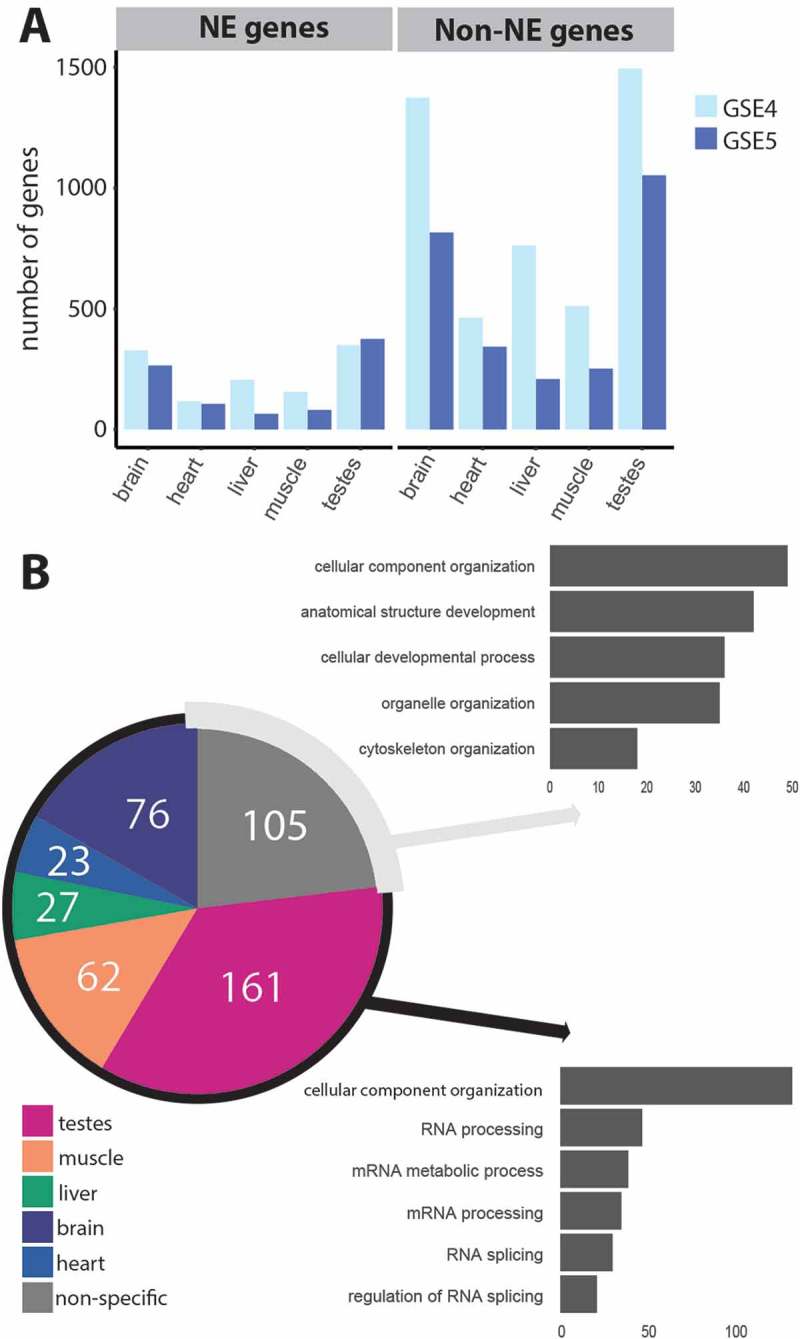
RNA support for novel tissue-specific splice variants through scoring of splice junctions according to their unique appearance in particular tissues. (a) The appearance of individual splice junctions in the GSE4 (light blue) and GSE5 (dark blue) tissue datasets was quantified for brain, heart, liver, muscle and testes. A scoring system (see text) for tissue-specificity was applied and the number of genes with tissue-specific splice junctions for each tissue is plotted. Brain and testes have the most specific isoform expression both when just considering genes encoding NE proteins and when considering non-NE protein genes. In total, 6,709 previously un-annotated differentially spliced junctions were identified for rat between the GSE4 and GSE5 datasets, 24% (1,622) of which were in NE genes. Many of these, however, were not found in both datasets. The intersection of the two datasets yielded 1,634 novel splice junctions, of which 28% (454) were in NE genes. (b) Most novel junctions in NE genes are tissue-specific and there seems to be a functional difference between those that can be defined as tissue-specific and those that are non-specified. The pie chart shows the tissue-specificity of the 454 ‘intersect’ un-annotated splice junctions in NE genes that were identified to be differentially spliced in both rat body map datasets. The graphs show the number of genes significantly enriched in GO term categories (p ≤ 0.01) as determined by LAGO analysis for tissue-specific genes (bottom) and non-tissue-specific genes (top).

To examine tissue-specific splice junctions we used the two rat body map datasets. In order to establish which splice junctions were tissue-specific a scoring system was designed in which E(dPSI) values for each tissue comparison were stored in separate tables for each tissue. Then, the E(dPSI) values were thresholded at a value of 0.2 such that a 1 indicates inclusion of the splice junction in the tissue described in the specific table and a 0 describes exclusion or no change. The scores were tallied and transferred to a separate table for comparison. A splice junction was defined to be tissue-specific when it had a higher final score for one tissue compared to all others tested. For example, a highly muscle-specific junction would have a muscle table score of 4 (the maximum number of comparisons possible with 5 tissues), and scores of 0 for liver, heart, testes and brain. Taking this very stringent definition of tissue-specificity, we found that, consistent with the literature, testes and brain had the most specific splice junctions and this was the case whether testing just NE genes or non-NE genes (Figure 3(a)).

To test if any of these NE un-annotated splice junctions were tissue-specific, we assigned the highest scoring tissue to each splice junction, and where two or more scores were tied we called the splice junction not specific (NA). In this way, 349 of the novel NE splice junctions are assigned as tissue-specific, with 105 not having a clear tissue specificity (Figure 3(b)). It might be that genes with these tissue-specific splice junctions have different roles to those where tissue-specificity is not as clearly defined. To check this we searched for gene ontology (GO) terms associated with each group using LAGO from the Princeton GO server [66] (http://go.princeton.edu/cgi-bin/LAGO). To our surprise the enrichment of GO terms was very different for the two groups. Genes containing novel splice junctions with a clearly defined tissue-specificity were enriched in GO terms concerning RNA processing, whereas genes that we could not classify in this way were enriched in GO terms related to structure and development (Figure 3(b)).

### Tissue-specific NE gene isoforms in genes relevant to nuclear envelope disease

In our analysis of the RNA-Seq data we identified novel variants for several genes relevant to nuclear envelope disease.

**Figure 4.**
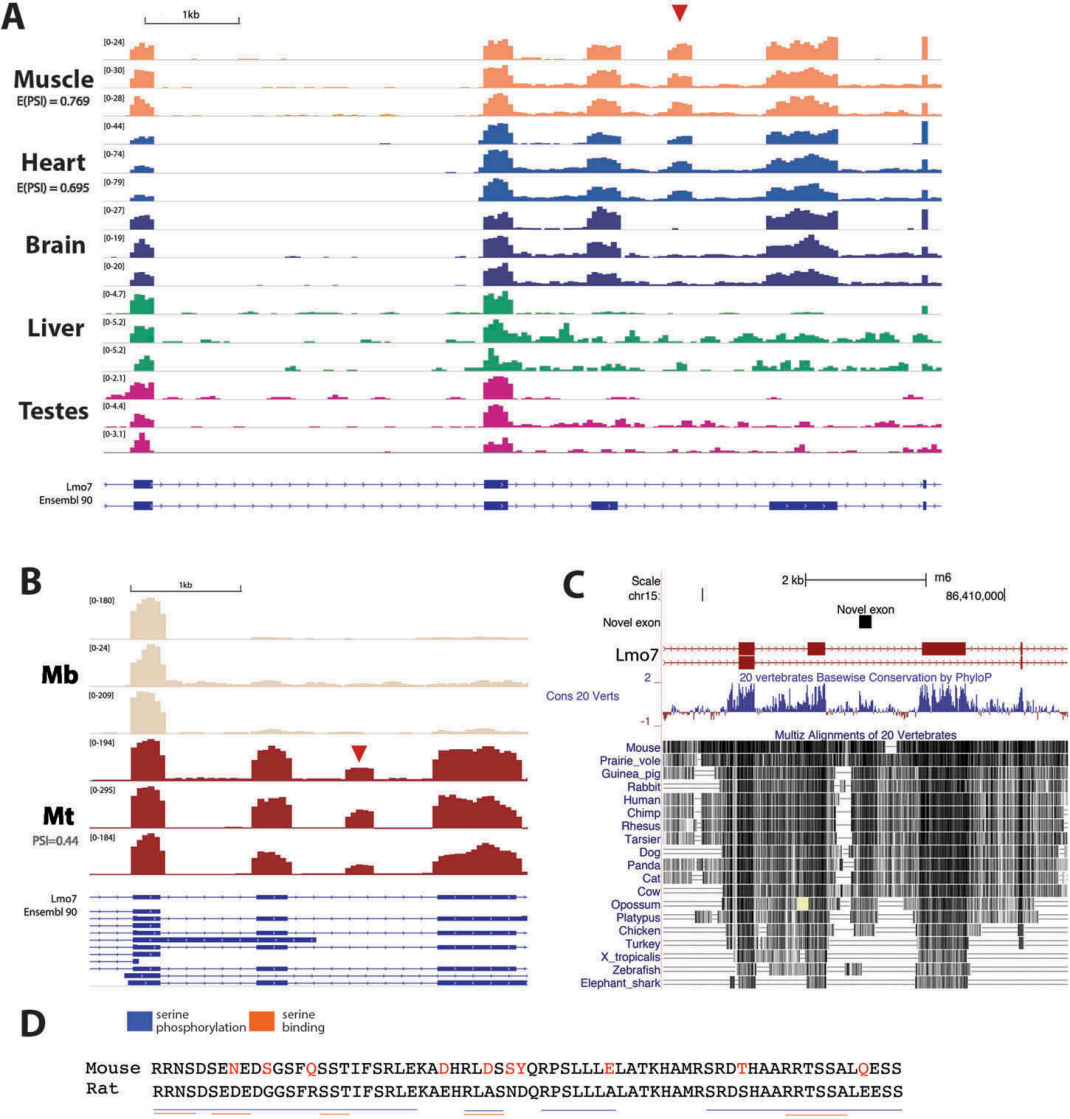
An unannotated exon in the regulator of emerin Lmo7 that is specific to heart and skeletal muscle. (a) Genome browser tracks show RNA-Seq read coverage over Lmo7, with the novel exon highlighted by a red arrowhead. GSE4 and GSE5 refer to two rat body map datasets produced by independent labs. E(PSI) values represent expected percent spliced in (PSI) values calculated by MAJIQ. (b) Again showing genome browser tracks, but for mouse C2C12 myogenesis data where each replicate is a representative sample from data from an independent lab. (c) PhyloP conservation track shows that the genomic region containing the novel exon is highly conserved between vertebrates. (d) Alignment of the mouse and rat exon amino acid sequences shows the high level of conservation, with non-conserved amino acid residues highlighted in red. Blue and orange lines indicate regions of the sequence where serine phosphorylation motifs and serine binding motifs respectively were identified by PhosphoMotif Finder.

*Lmo7 –* Emerin is an INM protein, that binds both lamin A and the BAF complex and is frequently mutated in Emery-Dreifuss muscular dystrophy. Lmo7 is a regulator of emerin [67]. We identified a novel exon in Lmo7 (mm10, chr14:101886131–101886331, Rn6, chr15:86407592–86407792) that we were unable to find described in any database. The exon is 201 bp long, and when spliced together with neighbouring exons is expected to be translated in-frame. There is evidence for inclusion of the exon in heart (E(PSI) = 0.695) and skeletal muscle tissue (E(PSI) = 0.769) in rat and differentiated myotubes in mouse (PSI = 0.44) across all datasets studied (Figure 4(a,b); note that the myogenesis junction was not detected by stringent MAJIQ analysis and so was calculated manually as described in the methods). Interestingly, a reasonable match to the exon appears in the gene sequence for all mammals and some avian species, but is notably absent in turkey, amphibians and fish (Figure 4(c)).

The expected amino acid sequence is serine-rich (Figure 4(d)) and so the sequence was searched for possible phosphorylation sites with PhosphoMotif Finder [68]. This software searches for the presence of sequences that have been identified in the literature, but does not perform statistical tests. In the rat sequence 8 possible phosphorylated serine binding motifs and 81 possible serine kinase/phosphatase motifs were predicted (Figure 4(d)). Many of these are in conserved regions of the sequence, for example the RRNS motif at the beginning of the sequence is a reported binding motif for 14-3-3 proteins, which are involved in cell signalling and transcriptional regulation [69]. The likelihood of high phosphorylation together with Lmo7 being only partly at the NE and thus of low abundance for recovered peptides and spectra readily explain its not being recovered in the mass spectrometry data.

**Figure 5.**
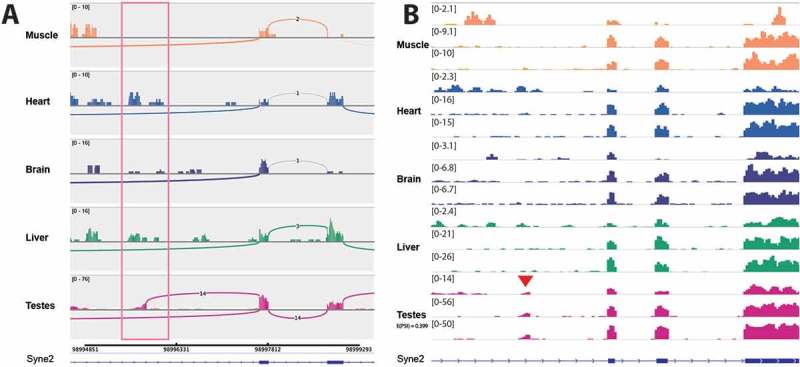
A novel testes-specific exon in Syne2. (a) Sashimi plot for GSE4 samples shows that even though the junction is not considered quantifiable by MAJIQ in this sample there are still reads spanning the junction. (b) Big wig files show GSE5 tracks as well.

*Syne2 –* The *SYNE* genes encoding nesprin proteins have been directly linked to Emery-Dreifuss muscular dystrophy [24]. We found transcriptome evidence for a testes-specific nesprin 2 isoform that is not annotated in human, mouse or rat Ensembl (E(PSI) = 0.4). It seems to represent a novel transcription start site, as it is only connected to the downstream exons (Rnor6 junction coordinate – chr6:98995844–98997669). Note that junction coverage in the region is especially poor: the junction is quantified by MAJIQ in the GSE5 sample due to the replicates, although there is still evidence for the junction in GSE4 (Figure 5(a,b)).

**Figure 6.**
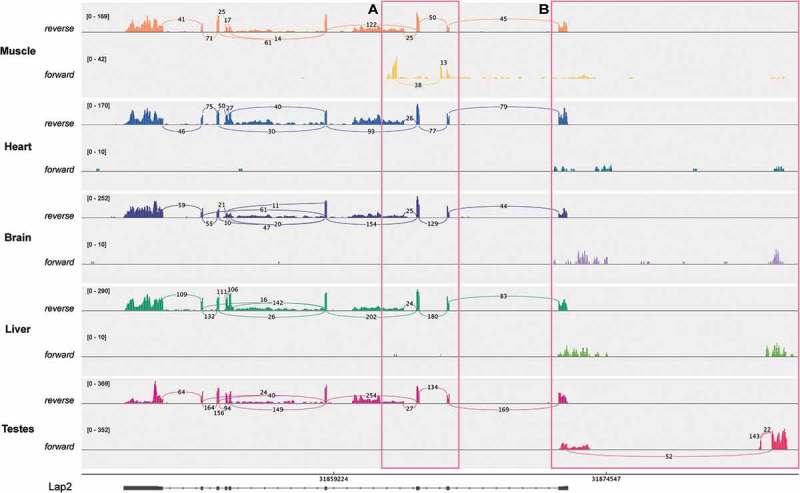
There is novel tissue-specific transcription in antisense to the Lap2 gene. Sashimi plots are shown for stranded GSE4 RNA-Seq samples, showing forward and reverse strand read coverage for each tissue studied. Tissue-specific transcription in antisense to the Lap2 gene is highlighted in pink boxes. (a) There is a muscle-specific transcription in antisense to Lap2 that is not annotated. (b) There is also transcription in antisense to the Lap2 start site, which is specific to testes. This transcript is not annotated in rat, however there is a similar transcript annotated in human.

*Lap2 (Tmpo) –* Lap2 is linked to dilated cardiomyopathy and hepatic steatosis [65,70]. In a potentially very exciting new aspect of Lap2 regulation, we discovered tissue-specific antisense transcripts in the Lap2 (*Tmpo*) gene that are confirmed by their appearance in both rat tissue datasets. This was incorrectly called as alternative splicing of Lap2 by MAJIQ analysis of the unstranded GSE5 dataset, thus highlighting the importance of using stranded RNA-Seq and checking potential splice variants in a genome browser. One internal antisense transcript is specific to muscle and another directed away from the transcription start site is specific to testes (Figure 6(a,b), respectively). The testes transcript is an annotated lncRNA in human and was identified from human prostate samples [71], but it is not annotated in mouse or rat and to our knowledge the tissue-specificity of this lncRNA has not been previously reported. Our discovery suggests that not only is this lncRNA conserved to rat, it is also highly tissue-specific and it seems likely it could be involved in regulating the expression of LAP2 isoforms in prostate and testis. The muscle-specific internal antisense transcript is located between exons 2 and 3. It is seemingly not annotated in any species and its function is unclear, however its proximity to the muscle specific LAP2α domain encoding exon 4 suggests that it could be involved in regulating this soluble isoform during myogenesis.

### Other potentially disease-relevant novel isoforms in muscle proteins

**Figure 7.**
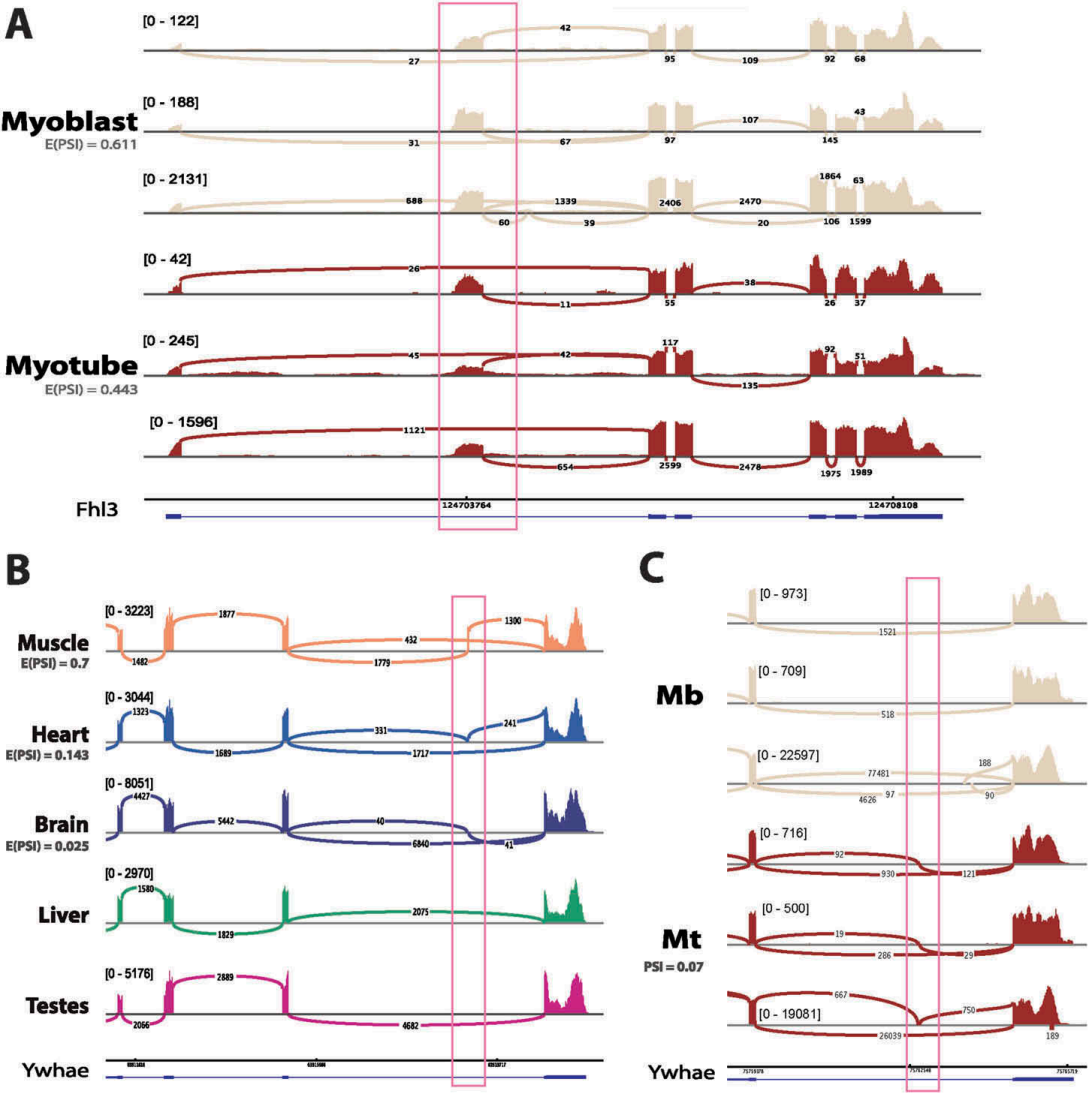
Additional novel splice variants with potential links to disease. (a) Novel alternative start in FHL3 detected in mouse C2C12 myogenesis. Sashimi plot shows raw coverage over junctions, with light blue denoting myoblasts and dark blue indicating myotubes. E(PSI) values are taken from MAJIQ analysis grouping together all samples from three independent labs. Mb: myoblast, Mt: myotube. (b) Ywhae has a muscle-specific exon in rat. Sashimi plot shows raw junction coverage over Ywhae for 5 rat tissues with one representative plot for each tissue, but the exon is observed in both rat tissue datasets. The blue transcript represents Ensembl 90 annotation. (c) The muscle-specific exon in rat is conserved in mouse and is specifically included in myotube. Mb: myoblast, Mt: myotube. Each track represents one sample from an independent lab for each condition.

FHL3 is closely related to FHL1 that is directly linked to Emery-Dreifuss muscular dystrophy[72]. FHL3 regulates the expression of muscle-specific genes in myogenesis, including MyHC [73]. In studying mouse myogenesis, we identified a novel exon in FHL3, that is present in both myoblast and myotube transcripts in RNA-Seq datasets from three independent groups (Figure 7(a)). Whilst the exon is included in both myoblast and myotube FHL3 transcripts, the inclusion isoform shifts from being the dominant isoform in myoblast (E(PSI) = 0.611), to being a minor isoform in myotube (E(PSI) = 0.443), suggesting that the change might be biologically relevant. We were unable to find any corresponding exon in rat tissue RNA-Seq, and there is no Ensembl annotation in rat, human or mouse for this exon. Given that the CDS begins in annotated exon 2, we expect this exon to be an extension of the 5′UTR and so could potentially be involved in regulating translation of the transcript.

Ywhae is a 14-3-3 protein that has been identified as a biomarker in mice with a limb girdle muscular dystrophy type E phenotype [74] and was identified in the NE proteomics datasets. We found evidence in the rat data for the possible muscle-specific expression of an exon in Ywhae that was previously only annotated in humans. This exon is highly specific to skeletal muscle tissue in rat (E(PSI) = 0.7) and is specifically expressed in myotube during mouse C2C12 myogenesis, although it is estimated to be present in only 7% of Ywhae transcripts (Figure 7(b,c). However, it also appears to be included to a much lesser extent in rat heart (E(PSI) = 0.143) and brain tissue (E(PSI) = 0.025). This is interesting because human diseases involving Ywhae tend to be heart or brain specific. For example, Ywhae has a role in neuronal migration, which is thought to be disrupted in Miller–Dieker syndrome [75]. While our data does not resolve full isoforms of Ywhae, there is only one annotated splice isoform in human containing this exon and it is a shorter isoform (732 bp, coding for 115 amino acids, where the longest annotated transcript is 2211 bp, coding for 255 amino acids), but there is no literature on the tissue-specific functions of this isoform. Our results argue that this splice variant is conserved to mouse and rat and is highly tissue-specific in its expression.

### Novel tissue-specific nucleoporin isoforms

**Figure 8.**
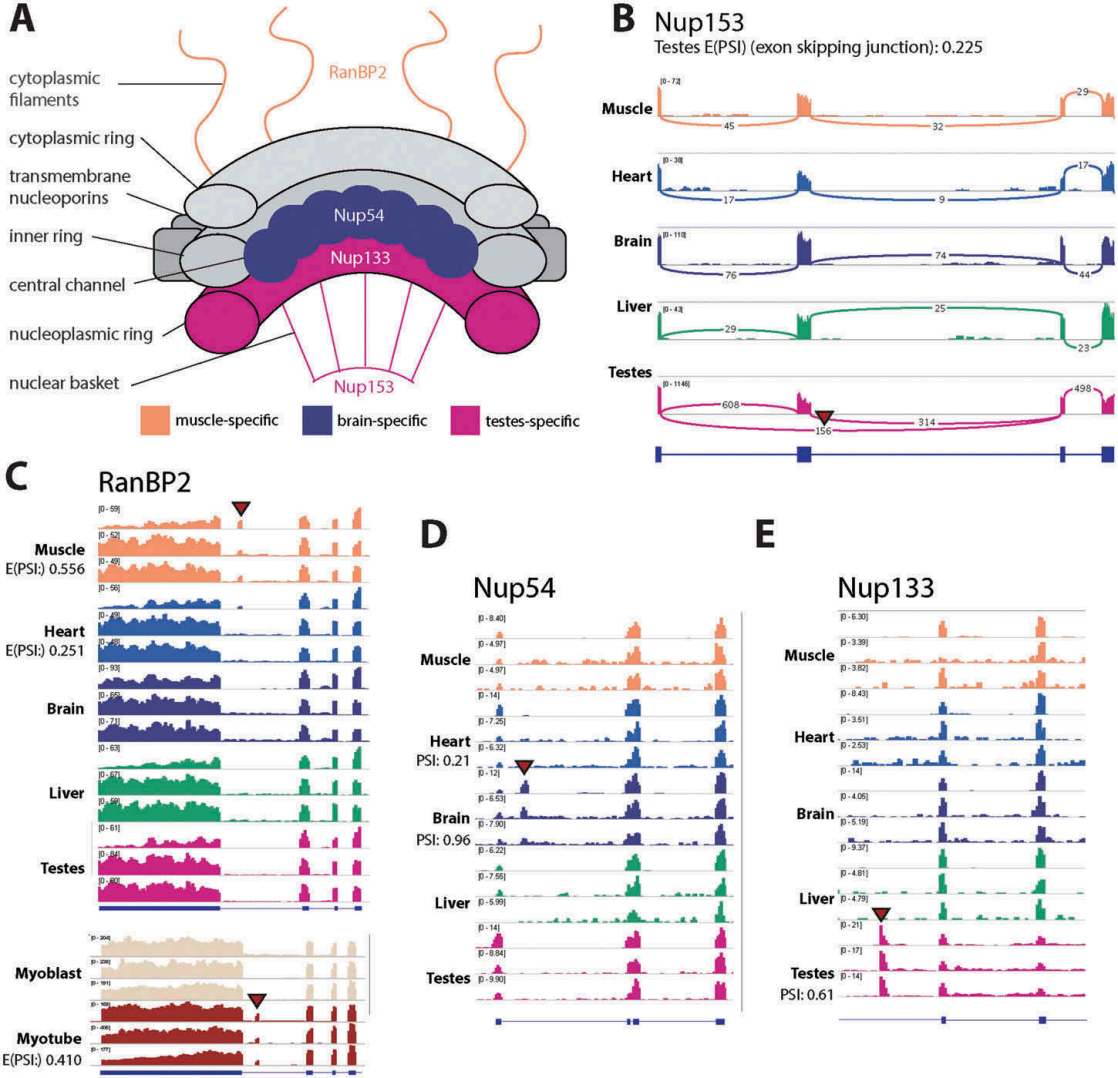
Multiple novel tissue-specific variants of nucleoporins were uncovered. (a) A schematic of the nuclear pore complex with novel tissue-specific splice variants annotated with their tissue-specificity. (b) For Nup153 a sashimi plot is shown to demonstrate the testes-specific exon skipping, the numbers over junctions are raw read coverage. (C-D) For RanBP2, Nup54 and Nup133 BigWig tracks are shown. For the rat data the top track for each tissue is GSE4, while the two below represent two replicates from GSE5. In the case of RanBP2 mouse C2C12 myogenesis data is also shown, where each track for the two conditions (Mb: Myoblast, Mt: myotube) is a representative sample from a dataset produced by independent labs.

We also identified novel tissue-specific isoforms in several nucleoporin proteins that are distributed throughout the nuclear pore complex (Figure 8(a)). These included a testes-specific exon skipping event in Nup153 (Figure 8(b); E(PSI) = 0.225) and a novel muscle-specific 78 bp exon in RanBP2 (Figure 8(c); E(PSI) = 0.556) that are not annotated in human, mouse or rat. A Nup54 brain-specific exon is annotated in human, but not mouse (Figure 8(d); PSI = 0.96), however to our knowledge it has not previously been reported that the exon is brain-specific. Furthermore, we found evidence for a novel testes-specific isoform in Nup133 (E(PSI) = 0.61).

The RanBP2 exon was also found to be specific to myotube in mouse C2C12 myogenesis (Figure 8(c), lower panel; E(PSI) = 0.410). The predicted amino acid sequence is located between a Ran binding domain and an E3 ligase domain and contains repeating proline and leucine residues with no premature termination codon. VastDB was checked for record of this exon and while no record was found for mouse, there is a 78 bp exon described for the human RanBP2 gene, which fits the inclusion profile observed in the current study. Taken together, there is very strong evidence for the conserved alternative splicing of this un-annotated cassette exon in vertebrate muscle.

### Functional validation of novel splice variants

**Figure 9.**
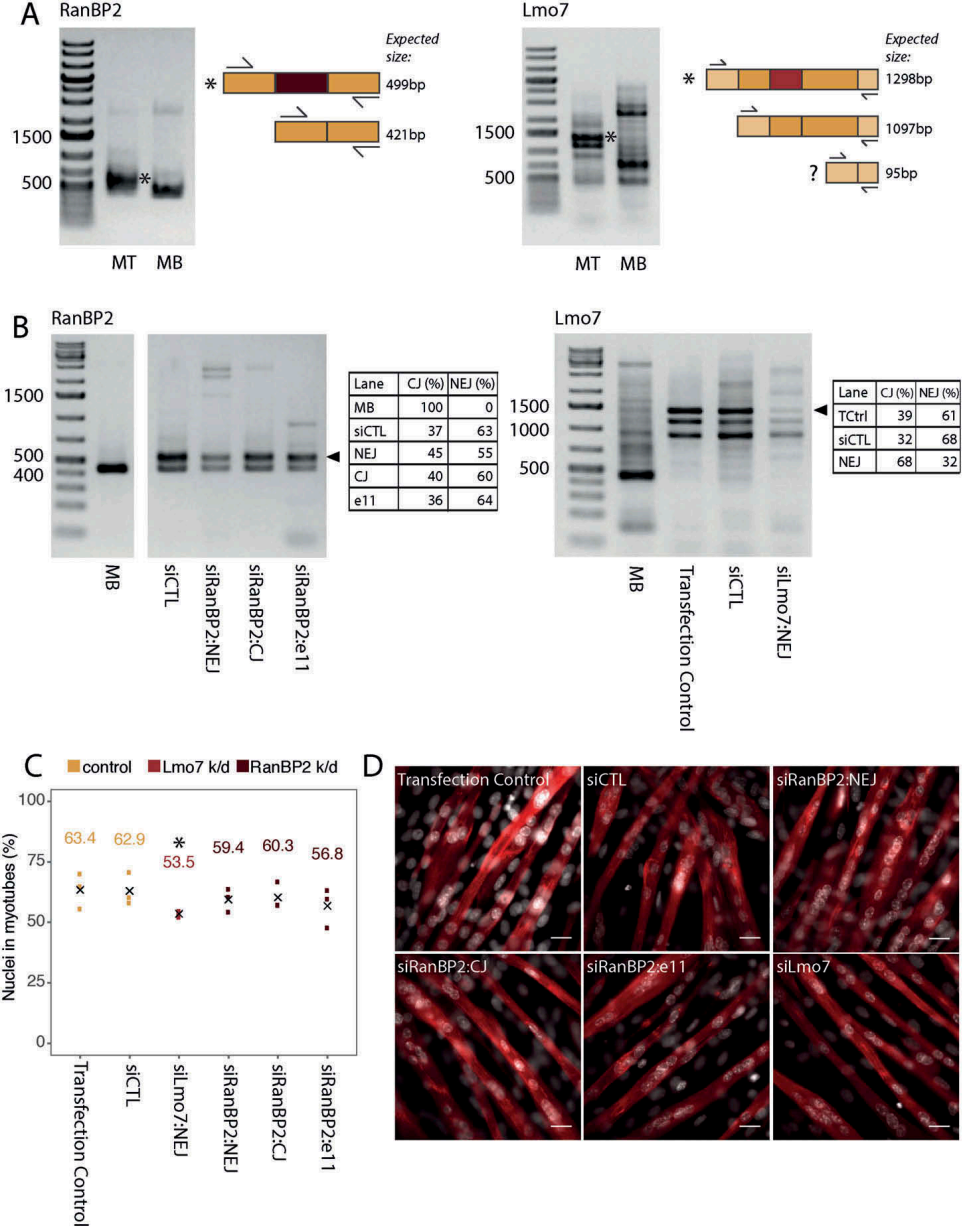
Functional validation of RanBP2 and Lmo7 novel splice variants in mouse C2C12 myogenesis. (a) RT-PCR for the novel myotube-specific exons reveals that they are spliced into mRNA produced in C2C12 mouse cells differentiated into myotubes (* marks novel splice form). Adjacent diagrams indicate the primer design and expected amplicon sizes. Note that for Lmo7 a third splice isoform is expected (marked with a question mark), however we don’t observe this in our gels (MB: myoblast, MT: myotube). (b) siRNAs against either the novel exon junction (NEJ), or canonical exon junction (CJ), or against a separate exon (e11), result in reduced mRNA expression of the specific RanBP2/Lmo7 splice variants. Novel splice variants are marked by an arrowhead. siCTL refers to treatment with a non-target control siRNA. Transfection control refers to treatment with the transfection reagents alone, but no siRNA. Tables next to each gel show the proportion of the novel and canonical isoforms in each quantifiable lane, as measured using the in-built gel analyzer tool in Fiji. (c) Quantification of myogenic index (percentage of nuclei in myotubes) as determined by myosin heavy chain 1 staining (red) in 4 day differentiated myotubes. The numbers above the points represent mean values from three biological replicates, the mean is also indicated with black crosses. * represents a statistically significant difference (p < 0.0001) between knockdown and non-target siRNA control conditions as measured using a Fisher’s exact test on the aggregated replicates. (d) Example fields of view used for determination of myogenic index in (c). Scale bars = 15 μm.

To validate some of the new splice variants in muscle development, mouse C2C12 myoblast cells were differentiated into myotubes in culture, RNA extracted, and analyzed by RT-PCR for the presence of the muscle-specific splice variants (Figure 9(a)). This clearly confirmed the expression of the novel Lmo7 and RanBP2 splice variants.

To assess potential functions of the tissue-specific splice variants they were specifically targeted by siRNAs in the C2C12 cells without affecting the canonical splice isoforms (Figure 9(b)). The myoblasts containing the siRNAs were induced to differentiate into myotubes so that the induced splice forms would be immediately targeted for degradation once they began to be expressed. By internally normalizing the signal from the novel isoform to the canonical isoform (novel/canonical+novel) we estimated that there was an 8% reduction in the novel RanBP2 isoform and a 36% reduction in the amount of the novel Lmo7 isoform in 4 day differentiated myotubes after treating with the specific siRNAs. Additionally, at day 4 the differentiated cultures were quantified for myogenic index by counting the number of nuclei in fused myotubes over the total number of nuclei in the population. We observed a reduction in successful fusion under the specific knockdown of the Lmo7 muscle-specific splice variant, in three independent biological replicates, with the difference ranging from 6–17% compared to a non-target siRNA control (Figure 9(c)). We aggregated the replicates and found the difference between specific and non-target siRNA treatment to be statistically significant (Fisher’s Exact Test, p-value < 0.0001), although due to the variability in the control conditions no firm conclusions can be drawn. Although there was no significant reduction in the myogenic index for the muscle-specific RanBP2 splice variant knockdown, images appeared to indicate some morphological differences such as in myotube thickness (Figure 9(d)) that may be of interest to further investigate in the future. Whether this or other subtle effects on differentiation are confirmed and can be linked to functional deficits such as mechanical function remains to be determined.

## Discussion

The original hypothesis for this study was that the use of tissue-specific organelle proteomics datasets would increase the likelihood of confirming translation of predicted tissue-specific splice variants both because the mass spectrometry data would be better matched and have greater depth from starting with an enriched fraction. While 183 unique junction-spanning peptides were identified, none of these turned out to be novel, due to poor rat genome annotations and this number reflects only a small fraction of the splice variants predicted from the RNA-Seq data. Whether this reflects a need for even greater depth in the mass spectrometry analysis or that most splice variants predicted by RNA-Seq data are not translated cannot be answered with the existing data, but several interesting observations nonetheless came from this work. First, much more splicing is conserved to rat and mouse in NE proteins than previously appreciated, suggesting important functional consequences for these splicing events. Moreover, alternate splicing is actually enriched in NE proteins as well as the presence of intrinsically disordered domains. This is consistent with ideas of NE proteins being involved in many different tissue-specific interactions and that alternative splicing is a means to modulate this. Intriguingly, many of the tissue-specific proteins are associated with RNA processing GO-terms, suggesting an unexpected role where tissue-specificity in the NE might propagate a wider cellular tissue-specificity through RNA processing. Most importantly, both the RNA-Seq and proteome analysis strongly support the prevalence of tissue-specific splice variants at the NE.

One such muscle-specific splice variant adds a novel exon in the regulator of emerin, Lmo7. Lmo7 acts as a transcription factor, activating the expression of emerin; however, Lmo7 also binds to emerin, reducing the pool of the protein available to act as a transcription factor and creating a negative feedback loop to downregulate emerin expression [67]. The predicted amino acid sequence of the novel exon is serine-rich and contains many possible phosphorylation sites, along with binding motifs for protein domains that bind phosphorylated serine residues. It seems likely that addition of this exon could confer a unique regulatory function to Lmo7 specifically in muscle tissues. Given its role in regulating emerin, this splice variant may be highly relevant for the study of Emery-Dreifuss muscular dystrophy (EDMD). In fact, the mild reduction in myogenic index is actually more strongly supportive of this idea than a block in myogenesis would be, because muscles develop and appear relatively normal before disease presentation typically when children become more active.

The finding of predicted novel alternative splicing events or novel antisense transcription in both other laminopathy-linked genes encoding nesprin 2 and Lap2 and other muscular dystrophy-linked genes such as FHL3 underscore the need for this type of analysis. When studying the function of genes relevant to muscular dystrophies, commonly genetic constructs are produced using commercially available cDNAs. Novel exons, such as those noted above, likely confer significant tissue-specific binding partners and/or regulation to these proteins. This could include loss of major partners under current investigation with commercial cDNAs or gaining of completely unknown partners that would only bind or be identified in pulldowns using the specific splice form. Regulation could include changes in protein post-translational modifications, cellular targeting, specific cleavage or overall protein stability. Therefore, it is important for researchers to use cDNAs that properly match tissue-specific sequences that could potentially be relevant to these diseases.

The novel tissue-specific exons for several nucleoporins uncovered raises a new and mostly unexpected mode of tissue-specific regulation at the NE – in control of protein trafficking. While some NPC components were known to have some tissue-specific variation in expression [31–34], the existence of tissue-specific splice variants was largely unexplored. This is not completely surprising in that transport receptors, particularly importin alpha, have long been known to have a variety of isoforms due to alternate splicing. The different isoforms are also consistent with a study indicating cell-type specific stoichiometries for NPCs [76]. This alternate splicing, as for the laminopathies, could contribute to tissue-specific disorders linked to these NPC proteins. For example, RanBP2 is implicated in many neurological conditions including Familial acute necrotizing encephalopathy (https://www.targetvalidation.org/target/ENSG00000153201/associations?view=t:table) caused by missense mutations, Parkinson’s disease due to its direct binding of the Parkin protein [77], and potentially Amyotrophic Lateral Sclerosis for which similar symptoms are achieved by RanBP2 knockout in mice [78]. RanBP2 is also associated with a wide range of cancers [18]. These data together argue that it is critical to identify all tissue-specific nuclear envelope splice variants and focus studies on using these specific isoforms in matched tissue systems. This study is a starting point and there is much more work needed to decipher the specific functions of these novel splice forms.

### Materials and methods

#### Datasets used

RNA-Seq from three mouse C2C12 myogenesis datasets and two rat body map datasets were used, summarized in Table 1.

**Table 1.** Summary of datasets used.

GEO Accession	GSE53960	GSE41637	GSE84158	GSE70389	GSE58928
Library Type	Single-end	Paired-end	Paired-end	Paired-end	Paired-end
	Unstranded	Stranded (dUTP method)	Unstranded	Stranded	Unstranded
	rRNA depleted but not poly(A) selected	Poly(A) selection	Total RNA	rRNA depleted, total RNA	rRNA depleted, total RNA
Read length	50bp	80bp	100bp	100bp	101bp
# Reads per sample	25–40 million, but 2 replicates were pooled to make 50–80 million	52–80 million	~15 million	37–76 million	142–161 million
# Biological replicates	8	1*	2 MB, 2 MT**	2 MB, 2 MT	1 MB 2 MT
Strain	Fischer 344	BN/SsNHsd (Brown Norway)	C2C12 mouse	C2C12 mouse	C2C12 mouse
Sex	4 female, 4 male	Male	-	-	-
Age	21 weeks	‘Breeding age’	MT – 7 day	MT – 4 day	MT – 4 day
Extra details	Skeletal muscle: gastrocnemius, ~100 mg ground Liver: ~100 mg ground Heart, brain, testis: Whole organ ground	Skeletal muscle: right quadriceps Heart: transmural Brain: visual cortex including grey and white matter Liver: unspecified Testis: Right, transverse section			Control from siRNA experiment, so treated with scrambled siRNA
Reference	(Yu et al., 2014) [54]	(Merkin et al., 2012) [56]	(Doynova et al., 2017) [79]	(Martone et al., 2016) [80]	(Singh et al., 2014) [81]

* There are three biological replicates per tissue, but two are sequenced to a much lower depth with 36nt read length and so were not used in the original paper for splicing analysis, and have been excluded from the present analysis due to MAJIQ’s dependence on junction reads for quantification.

** MB = myoblast, MT = myotube

#### RNA-Seq mapping and quality control

Reads were aligned to mouse (mm10) or rat (Rn6) genomes as appropriate using STAR (v2.4.2a) in two-pass mode [82]. Genome indexes were generated with Ensembl 89 annotations and produced independently for each mapping to avoid propagation of spurious alignments, although this could be considered conservative [83]. In the final stages junctions were compared against the latest Ensembl release (90) to ensure that they were truly novel. The median percentage of uniquely mapped reads across all samples used in the study was 85.51% (Max: 92.48; Min: 69.88). FastQC (v0.11.2) was used to check the quality of reads (https://www.bioinformatics.babraham.ac.uk/projects/fastqc/) and where necessary adaptors were trimmed using cutadapt (v1.9.1) [84] or Trim Galore! (v0.3.3) (https://omictools.com/trim-galore-tool) where quality trimming was also applied. rRNA regions were removed from bam files with BEDtools (v2.26.0) intersect -v, using a list of rRNA gene coordinates obtained from Ensembl BioMart [85,86]; additionally the 7SL gene was blacklisted as this region was over-represented in several samples.

For viewing many datasets in the Integrative Genomics Viewer (IGV) bigWig files were generated using deepTools (v2.5.1) bamCoverage with the flags – minMappingQuality 255 (to exclude multimapping reads) and – normalizeUsingRPKM so that tracks would be represented on a similar scale [87,88].

#### Splicing analysis

Differential usage of exon-exon junctions was quantified using MAJIQ (v1.0.3)[52]. Default settings were used with the critical criteria being – min_denovo 2, – minreads 3 and – min_intronic_cov 1.5 for the builder step, and – minreads 10 and – minpos 3 for the deltapsi step. Where a value is presented as E(PSI) this has been calculated using MAJIQ. Some novel events were identified outside of the MAJIQ analysis by screening genes of interest and in these cases PSI of a novel exon is calculated as: novel exon inclusion junctions (NEIJ)/(canonical exclusion junction (CEJ) * 2) + novel exon inclusion junctions (NEIJ) and reported in the text as PSI, rather than E(PSI). For myogenesis this value is presented as an average of three representative samples from the three datasets studied and in the case of the rat five tissue analysis this presents the value calculated from GSE4 samples.

For the rat body map data, datasets A and B were analyzed independently. The builder step was run on all tissue samples together and pairwise comparisons were made using the deltapsi module. Following this, E(dPSI) values for all splicing events in all tissue comparisons were combined into a single table. This table was processed using an R script to split splicing events into their component exon-exon junctions and assign a score ranking the inclusion of individual junctions in skeletal muscle, heart, liver, brain and testes tissues.

To score the inclusion of a junction in a specific tissue, tissue-specific tables were generated containing each possible comparison to that tissue and thresholded at E(dPSI) ≥ 0.2, where changes in PSI above this threshold were given a score of 1 and below this a score of 0. Scores were added for each tissue-specific table to give an overall tissue score, this gives an indication of the number of comparisons for which the junction was more included in the given tissue (Supplementary table 1).

For the mouse C2C12 myogenesis data all three datasets were analyzed together in MAJIQ, using the automatic weights feature to downweight outlier samples [89]. Splicing events with E(dPSI) ≥ 0.2 were considered to be differentially spliced. We checked our results against previously published studies to validate our approach (Supplementary Figure S4).

#### Enrichment analysis

To establish lists of expressed genes for each context, featureCounts was used to assign reads to gene-level counts and then DeSeq2 Variance Stabilizing Transformation (VST) was applied [90,91]. A calibration curve was produced where increasing cut off values were used to select expressed genes. Using this method there is a clear cut-off value where the group of genes selected decreases from all genes to a set of genes. The cut off was a normalized expression value of 3.1 for myogenesis and 3.8 for the rat body map data.

We supplemented this by also performing the analysis with genes that had an abundance of ≥1 transcripts per million (TPM)(GSE4: Supplementary Table 3A, GSE5: Supplementary Table 3B, C2C12 myogenesis: Supplementary Table 3C). Considering a gene’s count to be Ni and its length to be Li TPM was calculated as:

$$TPMi=10^{6}*\left(Ni/Li\right)/\Sigma j\;\left(Nj/Lj\right).$$

#### Intrinsic disorder analysis

The longest Ensembl annotated protein per gene was analyzed using IUPred (http://iupred.enzim.hu/). The number of amino acids with a score over 0.5 was recorded and number of proteins that had at least 20 such amino acids was counted.

#### Mass spectrometry analysis

The latest protein database for Rattus norvegicus (Rnor 6.0) was downloaded from Ensembl (ftp://ftp.ensembl.org/pub/release-90/fasta/rattus_norvegicus/pep/Rattus_norvegicus.Rnor_6.0.pep.all.fa) and exactly redundant sequences were removed keeping only one representative entry, leading to 27,229 non-redundant protein sequences. After adding usual contaminants (including keratins and proteases), all proteins entries were shuffled to estimate false discovery rates (FDRs), resulting in 54,836 amino acid sequences. Tissue-specific fasta databases for annotated and un-annotated splice junctions (192,897 and 177,043 amino acid sequences in the liver and muscle databases, respectively) were concatenated, 144,611 exactly redundant sequences were removed, and each NR protein entry was randomized (keeping the same amino acid composition and length). This annotated and novel junctions database was appended to the non-redundant Ensembl rat protein database described above to generate one database containing 252,554 rat proteins and junctions sequences, 193 contaminants, and 252,747 shuffled sequences. ProLuCID (v 1.3.3) [92] was used to search MS/MS datasets obtained from four NaOH-extracted and three Salt-and-Detergent extracted liver NE samples and four NaOH-extracted and two Salt-and-Detergent extracted liver NE protein samples. All protein samples had been digested with trypsin and analyzed by MudPIT as described previously [8,10]. To account for alkylation by chloroacetamide, 57 Da were added statically to cysteine residues, while 16 Da were added as a differential modification to methionines. Peptide/spectrum matches were sorted and selected using DTASelect/CONTRAST[93]. All 13 datasets were compared using CONTRAST: combining all runs, proteins had to be detected by at least 2 peptides and/or 13 spectral counts, leading to average low FDRs of 0.15% and 0.03% at the protein and spectral levels, respectively. To report all splice junction peptides passing selection criteria, proteins that were subsets of others were NOT removed using the parsimony option in DTASelect. Proteins that were identified by the same set of peptides (including at least one peptide unique to such protein group to distinguish between isoforms) were grouped together, and one accession number was arbitrarily considered as representative of each protein group. To estimate relative protein levels and to account for peptides shared between proteins, normalized spectral abundance factors (dNSAFs) were calculated for each detected protein, as previously described [94]. A full table of results is presented in Supplementary Table 2.

The original raw instrument files for these muscle and liver datasets may be obtained from the Massive repository: ftp://MSV000081166@massive.ucsd.edu and ftp://MSV000081167@massive.ucsd.edu, using MSV numbers as login and EH93JR as password.

#### Cell culture and transfection

C2C12 myoblasts (MBs) were cultured under ATCC-recommended conditions, except fetal bovine serum was used at a final concentration of 20%. MBs were induced to differentiate at 24 h post-confluency by removal of culture medium and the addition of DMEM with 2% horse serum. Differentiation media was replaced every 24 h up to 96 h post-induction. Because we have often observed loss of myotubes due to strong contractions that break ECM interactions, 1 μM tetrodotoxin was added at 72 h post-induction to inhibit contraction. Cells were transfected with siRNAs to specific splice variants as myoblasts using Lipofectamine 2000 (ThermoFisher) 24 h prior to starting differentiation induction. siRNAs used were as follows: siCTL Sense – AAUUCUCCGAACGUGUCACGUdTdT,

Antisense – ACGUGACACGUUCGGAGAAUUdTdT, siRanBP2:NEJ Sense -ACCUGAGAGCAAAGCACAAdTdT, Antisense – UUGUGCUUUGCUCUCAGGUdTdT, siRanBP2:CJ Sense – AGGCACAAGAGAAGAGCAAdTdT, Antisense -

UUGCUCUUCUCUUGUGCCUdTdT, RanBP2:e11 Sense – CCAGUCACUUACAAUUAAAdTdT (Note, this siRNA was used in a previous study[31]), Antisense – UUUAAUUGUAAGUGACUGGdTdT, Lmo7:NEJ Sense – CAGGGAUUCAGAAGGAAUUdTdT, Antisense -AAUUCCUUCUGAAUCCCUGdTdT (Sigma).

Differentiated myotubes were stained with myosin heavy chain 1 (Myh1) mouse monoclonal antibody clone My-32 (M1570 Sigma) together with 4,6-diamidino-2 phenylindole, dihydrochloride (DAPI). Images were acquired on a Nikon TE-200 microscope using a 1.45 NA 100x objective, Sedat quad filter set, PIFOC Z-axis focus drive (Physik Instrubments) and a CoolSnapHQ High Speed Monochrome CCD camera (Photometrics) run by Metamorph image acquisition software. Myogenic index was calculated as the percentage of total nuclei as assessed by DAPI staining that occurred within Myh1-stained myotubes in the population at 4 days post induction of differentiation. 5 fields across 3 biological replicates were counted to determine the myogenic index.

#### Reverse transcription PCR (RT-PCR)

cDNAs were generated using the Thermoscript II RNAse H – Reverse Transcriptase as per manufacturer’s instructions. Briefly, following annealing to a polyA primer at 65°C, 2 μg of total RNA was incubated with 1X Thermoscript RNAse H- reaction buffer, 10 U RNAsin, 10mM DTT, 1μM dNTPs mix and 200 U Thermoscript II RNAse H – Reverse Transcriptase. Tubes were vortexed, centrifuged briefly, and incubated at 42°C in a thermocycler with the heated lid at 80°C for 3 h. After heat inactivation of the reverse transcriptase by a 15 min incubation at 70°C, RNA was removed by incubating samples with 1 μL of 7 mg/ml RNAse A for 45 min at 37°C. cDNA was diluted with 90 μl of water and the reactions stored at −20°C.

For RT-PCR, 20 μl reactions containing 100ng of diluted cDNA and 1 μM forward and reverse primers were used. Primers for RT-PCR were designed using the Primer3 PCR web tool and were: RanBP2 forward primer 5′-CTGAAACAACACCAAAAGCAGT-3′, RanBP2 reverse primer 5′-GGGGTGGGAGTGAGTTCATA-3′, Lmo7 forward primer 5′ GTGCCAGCACCTTTGAGG-3′ and Lmo7 reverse primer 5′- CCATTCTCACCATAGATC-3′.

Bands from the RT-PCR were quantified using the in-built gel analyzer tool in Fiji [95]. The proportion of novel exon junction (NEJ) inclusion to canonical junction (CJ) inclusion was calculated by taking raw intensity NEJ/(raw intensity CJ + raw intensity NEJ) or vice versa for quantifying the CJ.

## Supplemental data

Supplemental date for this article can be accessed here.
