# Supplementary material for: Analysis of RNA-Seq datasets reveals enrichment of tissue-specific splice variants for nuclear envelope proteins
Source: Nucleus. 2018 Oct 6;9(1):410–30. doi: 10.1080/19491034.2018.1469351 (PMC7000147; doi:10.1080/19491034.2018.1469351)
Supplement: Supplemental Material [file kncl-09-01-1469351-s001.docx]

**Supplementary Material**

Supplementary tables

­­

Supplementary Table 1: Results of tissue-specific RNA-Seq analysis showing all unannotated NE junctions in rat Ensembl 90 annotations and their corresponding tissue-specificity. Additionally, the “in both” column denotes whether the junction was found in both GSE5 and GSE4, or just one.

Supplementary Table 2: Results of mass spectrometry search in NE data for rat muscle and rat liver.

Supplementary Table 3: Raw numbers for splicing enrichment calculations, including two measurements of expression (TPM and VST as described in the methods).

Supplementary figures

Figure S1. Need for alternative splicing analysis and data pipeline.

(**A**) Genome annotations likely remain incomplete based on increasing numbers of variants added to each subsequent annotation that have yet to plateau. Every Ensembl annotation release from 45 through to 90 was taken and the number of transcripts per NE gene was calculated using a custom bash script. With every subsequent annotation release more transcripts are annotated in mouse, with a dip after the release of a new genome assembly as it takes time to reconstitute the annotations. In rat the annotations are much poorer. (**B**) Schematic of pipeline used to identify novel splice forms from both RNA-Seq and NE proteomics data.


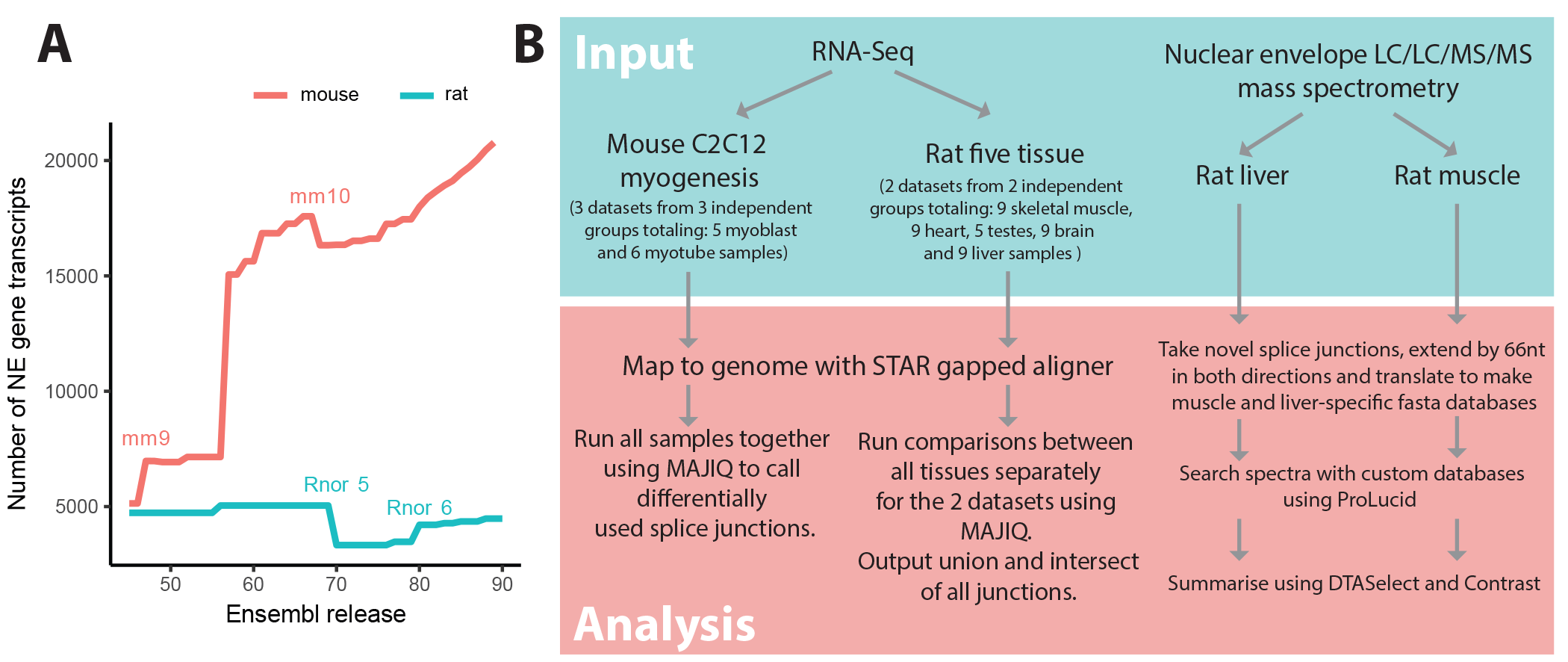


Figure S2. PCA analysis of myogenesis samples

To investigate the correlation between samples PCAExplorer was used to perform a principle component analysis (PCA) (Marini Federico, 2017). Firstly, gene-level counts were generated using featureCounts (Liao, Smyth and Shi, 2014). The counts were normalized using varianceStabilizingTransformation (VST) from the DeSeq2 package, blind to experimental condition (Love, Huber and Anders, 2014). C2C12 myoblast and myotube samples from three independent datasets cluster together appropriately.


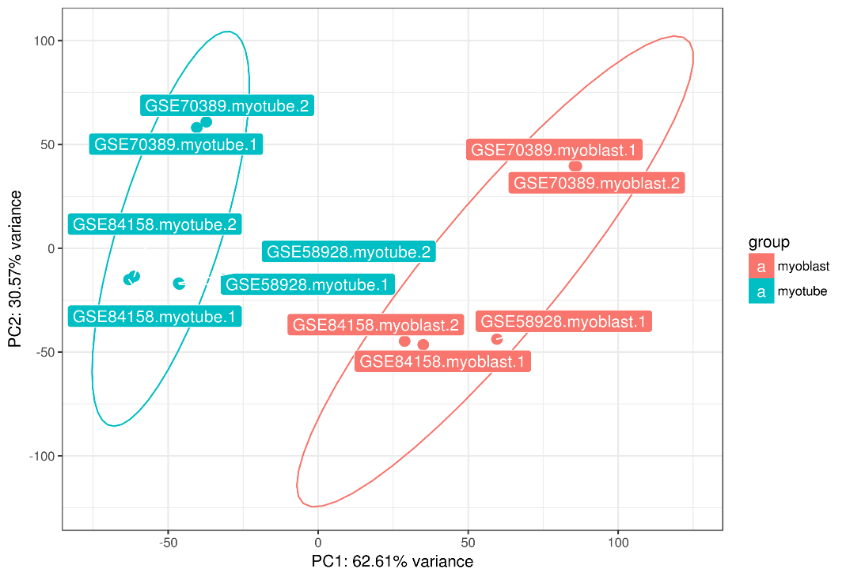


Figure S3. Annotated junctions rarely mark introns less than 60 nt long

Intron length was calculated for annotated and unannotated junctions and plotted as a histogram, shown here for a representative sample. Annotated introns are rarely shorter than 60 nt long.


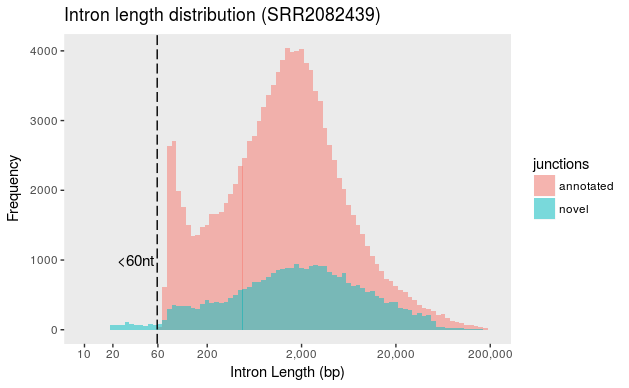


Figure S4. Comparison of myogenesis splicing analysis to previous studies

Genes called as differentially spliced were compared to previous studies of myogenesis to assess how well the current analysis corresponded to previous studies. Differences in quantification methods precluded a clustering analysis at the level of splicing events and so the comparison was made at the gene level. Our analysis was compared to two previous studies: a microarray study and a CuffDiff analysis of one dataset utilized in the present study (Bland *et al.*, 2010; Singh *et al.*, 2014). 44% of the genes identified as differentially spliced in the present analysis were also previously identified in the microarray and/or CuffDiff analysis, with 45 genes being common to all three analyses. Whilst there is a high level of similarity between the studies, validating the present approach, each study has unique splicing calls that may be due to confounding experimental variables or variation in computational analysis. As the present study combines data from multiple labs, the unique calls might represent splicing events that are weakly supported in individual experiments, but reach a quantifiable threshold in the combined analysis, but this was not explicitly checked.


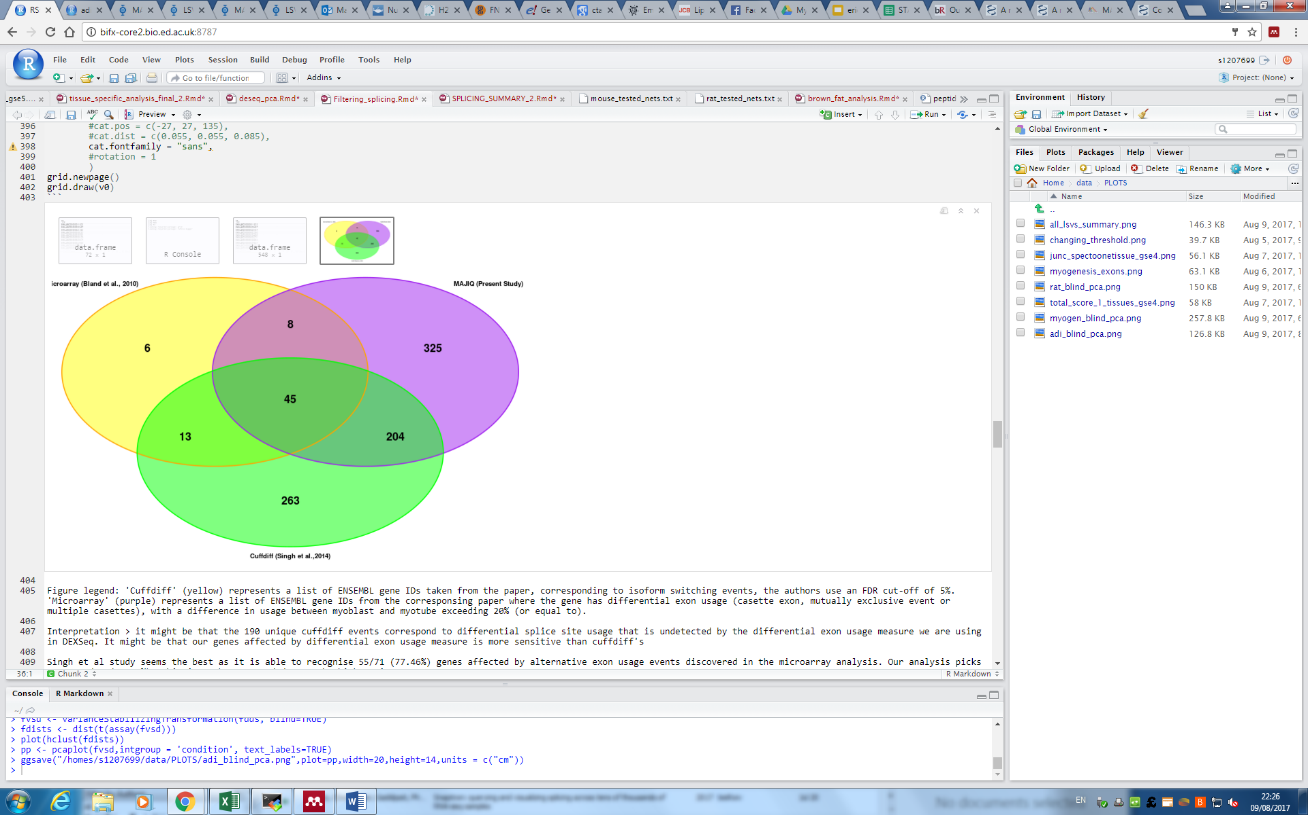


**Present study**

**Singh et al., (2014)**

**Microarray (Bland et al., 2010)**

References

Bland, C. S. *et al.* (2010) ‘Global regulation of alternative splicing during myogenic differentiation’, *Nucleic Acids Research*. Oxford University Press, 38(21), pp. 7651–7664. doi: 10.1093/nar/gkq614.

Liao, Y., Smyth, G. K. and Shi, W. (2014) ‘featureCounts: an efficient general purpose program for assigning sequence reads to genomic features’, *Bioinformatics*, 30(7), pp. 923–930. doi: 10.1093/bioinformatics/btt656.

Love, M. I., Huber, W. and Anders, S. (2014) ‘Moderated estimation of fold change and dispersion for RNA-seq data with DESeq2’, *Genome Biology*, 15(12), p. 550. doi: 10.1186/s13059-014-0550-8.

Marini Federico (2017) *pcaExplorer: Interactive Visualization of RNA-seq Data Using a Principal Components Approach*,  *R package version 2.2.0*. Available at: https://github.com/federicomarini/pcaExplorer (Accessed: 9 August 2017).

Singh, R. K. *et al.* (2014) ‘Rbfox2-Coordinated Alternative Splicing of Mef2d and Rock2 Controls Myoblast Fusion during Myogenesis’, *Molecular Cell*, 55(4), pp. 592–603. doi: 10.1016/j.molcel.2014.06.035.
